# Cancer-related neuropathic pain in out-patient oncology clinics: a European survey

**DOI:** 10.1186/1472-684X-12-41

**Published:** 2013-11-07

**Authors:** Cristina Garzón-Rodríguez, Leonidas Lyras, Luis Olay Gayoso, Juan M Sepúlveda, Epaminondas Samantas, Uwe Pelzer, Sarah Bowen, Chantal van Litsenburg, Mette Strand

**Affiliations:** 1 Service of Palliative Care, Institut Catalá d’Oncologia, Barcelona, Spain; 2 Pfizer, Athens, Greece; 3 Medical Oncology Radiotherapy Unit in Huca Oviedo, Gijon y alrededores, Spain; 4 Medical Oncology, Hospital Universitario 12 de Octubre, Madrid, Spain; 5 3rd Department of Medical Oncology, Cancer Hospital “Agii Anargiri”, Kaliftaki N. Kifissia, Greece; 6 Charité – Medical University of Berlin, Charité Comprehensive Cancer Center, Berlin, Germany; 7 Peasedown St. John, Bath, Somerset, UK; 8 Pfizer, Capelle a/d IJssel, The Netherlands; 9 Pfizer, Ballerup, Denmark

**Keywords:** Clinical oncology, Epidemiology, Neuropathic pain, Outpatients, Questionnaire

## Abstract

**Background:**

Although pain is frequently experienced by patients with cancer, it remains under-treated. The primary aim of this study was to estimate the prevalence of cancer-related neuropathic pain (CRNP) in patients with chronic pain who attended an outpatient clinic for standard care in Europe (irrespective of the reason or stage of the cancer). The secondary aims of this study were to characterise pain and cancer in patients with CRNP (including treatment) and to evaluate the usefulness of the painDETECT (PD-Q) screening tool to help physicians identify a potential neuropathic component of cancer-related pain.

**Methods:**

An observational, non-interventional, cross-sectional, multi-centre study of adult patients with cancer using patient and physician case report forms (CRFs). Patients with CRNP were identified by physicians’ clinical assessments after examining the completed PD-Q.

**Results:**

A total of 951 patients visiting outpatient clinics across Europe were enrolled in this study between August 2010 and July 2011. Of these, 310 patients (32.60%; 95% confidence interval 29.62, 35.58) were identified as having CRNP. Twenty-nine of 39 (74.4%) physicians who completed the CRF relating to the PD-Q considered it a useful tool to help detect CRNP in daily practice and 28 of 39 (71.8%) indicated that they would use this tool in the future for most or some of their patients. Data from physicians before and after review of the completed PD-Qs showed a shift in clinical opinion (either to positive CRNP diagnosis [yes] or negative CRNP diagnosis [no]) in respect of 142 patients; about half of which (74) were categorised with an initial diagnosis of unknown. Opinions also shifted from a no to a yes diagnosis in 10 patients and from a yes to a no diagnosis in 51 patients.

**Conclusions:**

Approximately one-third of adults with cancer experiencing chronic pain attending outpatient clinics as part of routine care were considered to have CRNP in the opinion of the physicians after considering scores on the PD-Q. While physicians did not consider the PD-Q to be a useful tool for all patients, shifts in diagnosis before and after the use of this tool indicate that it may help physicians identify CRNP, especially where there is initial uncertainty.

## Background

There were an estimated 3.2 million new cases of cancer and 1.7 million cancer-related deaths in Europe in 2008 [1]. A systematic review of the prevalence of pain in patients with cancer was estimated at >50% (when cancer types were pooled); the highest prevalence of pain was reported in patients with head/neck cancer (70%; 95% confidence interval [CI] 51%, 88%) [2].

Although pain is frequently experienced by patients with cancer, it can remain under-treated [3,4] as a result, in part, of patients’ reluctance to report pain or to take treatment for pain relief in addition to cancer treatment [5,6]. Under-treatment can also be caused by a limited knowledge and practice of the treatment of pain among oncologists [5,6].

Identifying the type and source of pain in patients with cancer is complex. Sources of cancer pain vary from direct tumour invasion of bone, nerves, ligaments, etc.; metastasis of the disease; or side effects of treatment (chemotherapy, surgery etc.) [7]. Approximately 33% of cancer survivors were reported to experience continued pain [2]. Complex chronic pain syndromes can arise either from surgery, co-morbid conditions, recurrent cancer and/or treatment [8,9].

Cancer-related pain can either be nociceptive (musculoskeletal, cutaneous or visceral) or neuropathic. Neuropathic pain is defined by the International Association for Study of Pain as “pain caused by a lesion or disease of the somatosensory nervous system” [10]. Patients can experience nociceptive and neuropathic pain at the same time [11,12]. Establishing the nature of the pain in patients with cancer is key in providing effective pain relief because particular analgesics do not effectively manage both neuropathic and nociceptive pain. Between 19% and 39% of patients with cancer are believed to experience pain with a neuropathic component, many of whom experience a mixed pain condition [4]. Neuropathic cancer pain is associated with poorer physical, cognitive and social functioning and greater requirements for pain medications than nociceptive cancer pain [13]. Finding effective tools to enable physicians to better identify cancer-related neuropathic pain (CRNP) in an outpatient setting would therefore allow a greater number of patients to receive effective treatment for CRNP.

In the current study, in addition to physician assessment of neuropathic cancer pain, we used the painDETECT questionnaire (PD-Q), a validated screening tool that demonstrated high sensitivity and specificity for the detection of neuropathic pain (85% and 80%, respectively). [13,14]. The PD-Q is an easy-to-use, self-reported questionnaire that is available in a variety of languages.

The primary objective of this non-interventional study was to estimate the prevalence of CRNP in patients with cancer that experienced chronic pain and attended outpatient oncology clinics and palliative care services in Europe. The secondary objectives of this study were to calculate the prevalence point estimate of chronic cancer pain. The usefulness of the PD-Q screening tool [14] also was assessed as an aid for physicians to identify the neuropathic component of cancer-related pain. A further objective was to describe the characteristics and impact of pain and cancer in outpatients experiencing neuropathic pain.

## Methods

### Study design

This was an observational, non-interventional, cross-sectional, multi-centre study of adult patients with cancer experiencing chronic pain. The study was conducted between 23 August 2010 and 22 July 2011 in outpatient oncology clinics in Denmark, Germany, Greece, Spain and the UK. One of the clinics in Spain was a palliative care clinic. Data from 63 physicians from 49 sites were collected in one consultation only (no follow-up) when patients visited the doctor as part of standard practice. Data regarding diagnosis and disease management were collected retrospectively. Where appropriate, the protocols were reviewed and approved by institutional review boards/independent ethics committees at participating sites. Denmark had general permission to conduct non-interventional studies from the Danish Data Protection Agency (Datatilsynet) and did not need approval by the Danish Medicines Agency/Ethics Committee.

### Participants

Participants were adults aged ≥18 years who had chronic cancer-related pain (defined as pain most days of the week for ≥3 months), attended the oncology unit for an outpatient consultation (irrespective of the reason or the stage of the cancer) and were willing to provide informed consent. Patients with chronic pain that was believed by the physician to be unrelated to cancer were excluded from the study.

### Study enrolment

To obtain a representative sample of the oncology outpatient population seeking treatment for chronic pain in Europe, patients who attended oncology clinics from several centers in selected European counties were recruited. There was a flexible recruitment period to allow each physician to consecutively enrol up to 20 patients with chronic pain. In order to minimize any potential recruitment bias, identification and recruitment of patients eligible for the survey was consecutive. Each and every patient attending the outpatient clinic was considered for participation in the survey at the time that the patient consulted the doctor. In general, prevalence is calculated by dividing the number of individuals with a certain condition at a particular time point by the total number of individuals seen. It is therefore important to ensure that the inclusion of suitable patients by the doctor following recruitment of the first patient is consecutive, in order to estimate a valid prevalence of chronic pain in patients attending oncology clinics as well as the prevalence of CRNP in this population. The time of recruitment was recorded for all patients included in the survey and the prevalence was then calculated. The protocol was amended in May 2011 to allow the continued recruitment of patients by an additional physician at the same site (up to a maximum of 40 patients, with sponsor permission) if a particular physician was no longer able to participate in the study.

Eligible participants were identified and screened for chronic pain (defined in the Participants section above) by study physicians during consecutive appointments to minimise potential recruitment bias (i.e. every patient visiting the oncologist during the recruitment period was considered for participation in the study). A screening log was used to register all patients visiting the physician on an outpatient-basis during the consecutive screening and enrolment period. As some patients attending the clinics may have been receiving pharmacological treatment to manage their pain, the screening log also was intended to enable physicians to identify which patients would have been experiencing chronic pain had their pain not been effectively managed with pharmacological treatment.

### Study procedures

**Figure 1:**
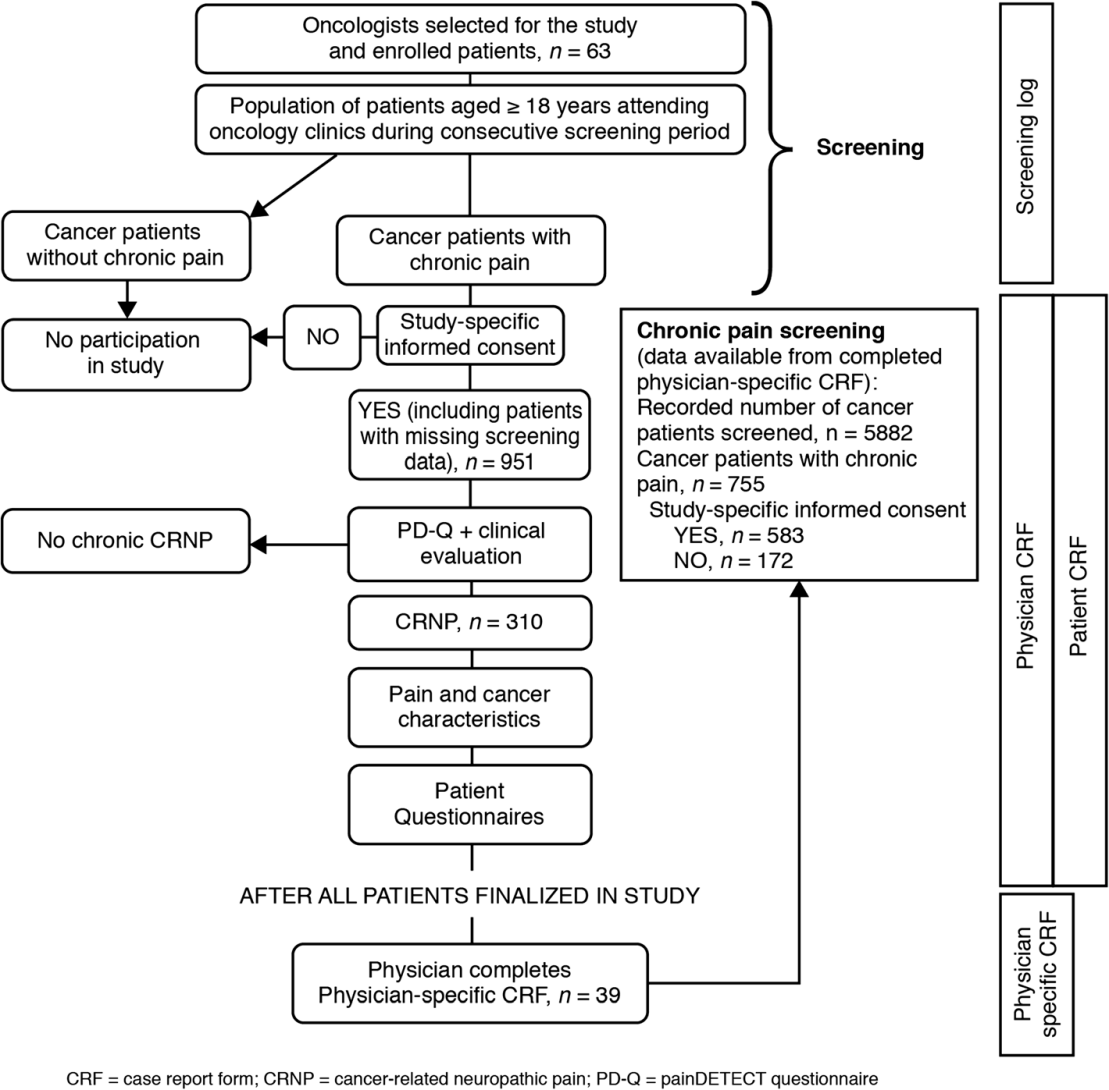
Procedure flow.

A summary of the study procedure is shown in Figure 1. Eligible participants enrolled in the study completed the PD-Q, which was used in conjunction with physician assessment to identify patients with chronic pain who also experienced CRNP [14]. Patients with CRNP were identified based on the clinical opinion of the physicians before and after considering scores on the PD-Q.

Physicians and patients with CRNP completed case report forms (CRFs) to record the characteristics of cancer and pain in the CRNP population. Questions on the CRFs related to the history and therapeutic management of cancer (types of intervention, since time of diagnosis) and neuropathic pain (duration, aetiology, pharmacological treatment/s for pain, etc.). Patients with CRNP also completed a series of self-reported questionnaires and rating scales to assess the impact of their symptoms and pain.

After all patients completed the study, physicians were asked to complete a physician-specific CRF relating to the usefulness and future use of the PD-Q and prevalence data.

#### painDETECT questionnaire (PD-Q)

The PD-Q is a validated screening tool [14] developed to assess the characteristics, persistency, frequency and location of presenting pain on numerical scales.

Patients responded to three preliminary questions that did not contribute to the PD-Q end score to document the intensity of their pain using a 0–10 rating scale.

The description of pain (persistent with slight fluctuations [score = 0], persistent with pain attacks [score = 1], or pain attacks without pain between them [score = 1]) was recorded along with the presence (score = 2) or absence (score = 0) of radiating pain.

The nature of pain was assessed in a series of seven questions (e.g. is light touching [clothing, a blanket] in this area painful? Do you suffer from a burning sensation [e.g. stinging nettles] in the marked areas?). Responses were recorded on a 0–5 rating scale (never = 0, hardly noticed = 1, slightly = 2, moderately = 3, strongly = 4, very strongly = 5).

PD-Q scores relating to the description and presence of radiating pain (maximum score of 3) and the nature of pain (maximum score of 35) were summated into a maximum PD-Q end score of 38. Higher end scores indicated a greater likelihood of neuropathic pain (end scores <13 = unlikely, 13–18 = possible, >18 = likely).

#### modified Brief Pain Inventory Short Form (m-BPI-sf)

The severity and interference of pain was assessed via the self-reported numeric rating scales (range, 0–10) of the m-BPI-sf [15].

#### EuroQoL Health Questionnaire (EQ-5D)

Health-related quality of life was assessed in relation to: mobility, self-care, usual activities, pain/discomfort, anxiety or depression on a three-point response scale of the EQ-5D Health Questionnaire [16] combined into a single index utility score (range, 0–1). Patients’ own perception of their health (how good or bad) also was assessed on this questionnaire via a self-reported numeric rating scale (health state score; range, 0–100).

#### The Sheehan Disability Scale (SDS)

The level of functional impairment in relation to work or school, social life and home life and family responsibilities was recorded on the SDS [17] using self-reported 10-point visual analogue scales and summated into a global functional impairment score (maximum 30 = highly impaired). Patients also recorded the number of lost and underproductive days of work during the past week on this form.

### Outcome measures

The primary endpoint was the proportion of patients experiencing chronic pain that were considered to have CRNP in the clinical opinion of the physicians following an examination of the completed PD-Q.

The point estimate of the proportion of participants experiencing chronic pain was calculated from the total number of participants screened. However, owing to design flaws in the screening log, the information relating to patients whose chronic pain was controlled with pain medications was not reliably transferred to the physician-specific case report form (CRF). Consequently, the chronic pain calculations excluded patients who did not meet the chronic pain criteria owing to the use of pain medications.

Other secondary endpoints included a description of the nature, characteristics and management of cancer and pain in patients with CRNP and the percentage of cancer specialists who found the screening tool a useful instrument to identify chronic neuropathic pain in daily practice.

### Statistical analysis

#### Sample size and study populations

The projected sample size was changed from approximately 3900 patients to at least 800 patients in May 2011 owing to difficulty finding sites and physicians who were willing to take part. However, a sample size of at least 800 patients was still estimated to reliably identify the primary endpoint (prevalence of CRNP) based on the previously estimated prevalence of 39.7% [18]. The pre-defined study populations were as follows: 1) the all participants population, defined as all those enrolled in the study. 2) the CRNP population, defined as those patients identified as experiencing neuropathic pain in the clinical opinion of the physicians after examining scores on the PD-Q; and 3) the surveyed physicians population, defined as those physicians who completed the physician-specific CRF.

#### Data analysis

Data used to estimate the prevalence of CRNP for all participants enrolled had an estimated precision of 0.03 (3%; using a 95% CI). Continuous endpoints were summarised using descriptive statistics and discrete endpoints were summarised using frequency and percentage calculations for each response category (missing data were excluded from the percentage calculations).

## Results

### Patient and surveyed physician populations

A total of 951 patients were enrolled in the study and 937 (98.5%) patients completed all or part of the study (Figure 1). Four (0.4%) patients withdrew during the screening phase as they were no longer willing to participate in the study. Data for 10 (1.1%) patients were either missing owing to an entirely missing CRF or a missing final status page from the CRF.

A total of 63 physicians enrolled patients for the study; 39 of these also completed the physician-specific CRF (Figure 1).

Missing data resulting from the missing CRFs or missing CRF pages in this study are reported for each of the endpoints in the relevant sections or tables.

### Patient demographics

The age ranges of the all participants population are shown in Table 1. Male participants were slightly older, on average, than female participants.

**Table 1:** Demographic characteristics of patient populations

	**Male**	**Female**	**Participants for whom sex was not recorded**^**b**^	**Total**
All participants^a^, n	502	432	11	945
Age, years, n (%)				
18-44	17 (3.39)	39 (9.03)	2 (18.18)	58 (6.14)
45-64	189 (37.65)	220 (50.93)	5 (45.45)	414 (43.81)
≥65	296 (58.96)	173 (40.05)	1 (9.09)	470 (49.74)
Missing data	0	0	3 (27.27)	3 (0.32)
Mean (SD)	65.88 (11.34)	60.94 (12.33)	50.75 (13.05)	63.48 (12.12)
Patients with CRNP^a^, n	144	155	9	308
Age, years, n (%)				
18–44	4 (2.78)	20 (12.90)	2 (22.22)	26 (8.44)
45–64	60 (41.67)	88 (56.77)	4 (44.44)	152 (49.35)
≥65	80 (55.56)	47 (30.32)	1 (11.11)	128 (41.56)
Missing data	0	0	2 (22.22)	2 (0.65)
Mean (SD)	64.67 (10.31)	58.08 (12.35)	50.14 (13.97)	61.00 (12.01)

^a^Incorrect dates of birth were recorded for six patients in the all participants population and two patients in the CRNP population.

^b^Age data was missing for three patients in the all participants population and two patients in the CRNP population in those patients whose sex was unknown.

CRNP, cancer-related neuropathic pain; SD, standard deviation.

### Primary endpoint

Of the 951 patients identified to have chronic pain, 310 patients were considered to experience CRNP in the clinical opinion of the physicians after examining scores on the PD-Q. Thus, the prevalence of CRNP (primary endpoint) was 32.6% (95% CI 29.62, 35.58). The number of patients considered to experience CRNP by the physicians before examining PD-Q scores was 335 (35.2%; 95% CI 32.19, 38.26). The sex and ages of patients with CRNP were proportionally similar to the all participants population (Table 1).

### Secondary endpoints

#### Prevalence of chronic pain

Data from physicians invited to take part in the study who did not record the number of patients screened were not included in the prevalence of chronic pain analysis. The number of patients included in the chronic pain calculation differed from the 951 patients identified for calculating the primary end point. Of 5882 patients screened by 39 physicians (and for whom screening was documented), 583 met the criteria for chronic pain and were enrolled in the study and 172 patients met the criteria and declined participation (Figure 1). The prevalence point estimate of chronic pain was therefore calculated at 12.8% using the following equation:

583+172^*100/5882=12.8%

#### Nature and treatment of neuropathic pain in patients with CRNP

In the physicians’ assessment of the type of chronic cancer-related pain, 100 (32.3%) patients were considered to have pure neuropathic cancer-related pain and 146 (47.1%) patients were considered to have mixed cancer-related pain with a neuropathic component. Data were missing for 64 (20.6%) patients.

While chronic pain was experienced by all participants for >3 months, the duration of neuropathic pain in the CRNP population varied from <3 months to >3 years. Only 28 (9.0%) patients with CRNP reported having the pain for <3 months, with the greatest percentage (n = 114; 36.8%) having had the pain for 3–6 months. Fifty-five (17.7%) patients with CRNP reported having had the pain for 7–12 months, 45 (14.5%) patients had the pain for 13 months–3 years and 10 (3.2%) patients had the pain for >3 years. Data were missing for 58 (18.7%) patients.

In the opinion of the physicians, the neuropathic pain experienced by patients with CRNP was due to the tumour itself in 197 (63.6%) patients and due to cancer-treatment in 90 (29.0%) patients. Chemotherapy and surgery were each believed to account for neuropathic pain in 41 (13.2%) patients, respectively. The most commonly prescribed therapeutic treatments (previous and current prescriptions) for neuropathic pain in patients with CRNP were non-opioid analgesics, a strong opioid and/or anticonvulsants (Table 2).

**Table 2:** **Therapeutic management of neuropathic pain**^**a **^**in patients with CRNP**

**Prescribed treatment for neuropathic pain,**	**Previous**	**Current**
**n (%)**	**n = 310**	**n = 310**
Non-opioid analgesics	164 (52.90)	123 (39.68)
Weak opioid	62 (20.00)	35 (11.29)
Strong opioid	136 (43.87)	121 (39.03)
Antidepressants	36 (11.61)	29 (9.35)
Anticonvulsants	95 (30.65)	118 (38.06)
Muscle relaxants	11 (3.55)	7 (2.26)
Corticosteroids	44 (14.19)	44 (14.19)
Antispasmodics	2 (0.65)	0
Anxiolytics	41 (13.23)	30 (9.68)
Other	20 (6.45)	23 (7.42)
None	6 (1.94)	22 (7.10)

^a^More than one treatment type was possible for each patient.

CRNP, cancer-related neuropathic pain.

#### Pain-related characteristics of CRNP

Numerical rating scores for patients with CRNP were higher (indicating worse pain) on average compared with the all participants group for each of the following three preliminary PD-Q questions to assess pain: 1) “How would you assess your pain now, at this moment?”; 2) “How strong was the strongest pain during the past 4 weeks?”; and 3) How strong was the pain during the past 4 weeks, on average?” (Table 3).

**Table 3:** Patients’ assessment of pain: painDETECT scale 0 (no pain) to 10 (maximum pain)

**Question**		**Patients with CRNP**	**All participants**
		**n = 310**	**n = 951**
How would you assess your pain now, at this moment?	Mean (SD)	4.36 (2.58)	3.74 (2.55)
	95% CI	4.07, 4.65	3.57, 3.90
	Missing data, n	4	22
How strong was the strongest pain during the past 4 weeks?	Mean (SD)	7.94 (1.96)	7.23 (2.40)
	95% CI	7.72, 8.16	7.08, 7.39
	Missing data, n	3	20
How strong was the pain during the past 4 weeks, on average?	Mean (SD)	5.55 (2.00)	4.97 (2.06)
	95% CI	5.33, 5.78	4.83, 5.10
	Missing data, n	4	24

CI, confidence interval; CRNP, cancer-related neuropathic pain; SD, standard deviation.

Mean subscale scores on the m-BPI-sf for the CRNP population were 5.48 (95% CI 5.17, 5.80, n = 252) for pain interference and 4.63 (95% CI 4.38, 4.89; n = 256) for pain severity based on numerical rating scales of 0 (no pain) to 10 (pain as bad as you can imagine). Data were missing for 58 and 54 patients for pain interference and pain severity, respectively. Mean scores for individual items on the m-BPI-sf in the CRNP population were all between 4.08 and 6.58, with the exception of pain at its least in last 24 hours (mean = 2.92; Table 4).

**Table 4:** Summary of self-reported pain m-BPI-sf scales 0 (no pain) to 10 (maximum pain) in patients with CRNP

	**n**	**Mean (SD)**	**95% CI**	**Missing data, n**
Sub-questions of severity scale				
Pain on average in last 24 hours	256	4.96 (2.12)	4.70, 5.22	54
Worst pain in last 24 hours	256	6.58 (2.61)	6.26, 6.90	54
Pain right now	256	4.08 (2.66)	3.75, 4.41	54
Pain at its least in last 24 hours	256	2.92 (2.20)	2.65, 3.19	54
Sub-questions of interference scale				
General activity	256	5.91 (3.03)	5.53, 6.28	54
Mood	256	5.69 (3.02)	5.32, 6.06	54
Walking ability	256	4.98 (3.43)	4.55, 5.40	54
Normal work	254	6.35 (3.16)	5.96, 6.74	56
Relations	255	4.55 (3.27)	4.15, 4.95	55
Sleep	255	5.04 (3.34)	4.62, 5.45	55
Enjoyment of life	256	5.86 (3.29)	5.46, 6.27	54

CI, confidence interval; CRNP, cancer-related neuropathic pain; m-BPI-sf, modified Brief Pain Inventory Short Form; SD, standard deviation.

#### Characteristics of cancer and treatment in patients with CRNP

##### Types and characteristics of cancer

The types and characteristics of cancer in patients with CRNP are summarised in Table 5. The most common types of cancer in this population were breast cancer (18.1%) and lung cancer (14.5%). Many (44.2%) patients with CRNP had loco-regional progression of cancer and approximately half (49.0%) experienced metastasis of the disease in one or more site (Table 5). Bone was the most commonly reported site for metastasis (Table 5).

**Table 5:** **Summary of characteristics and type of cancer**^**a **^**in patients with CRNP**

**Characteristic**	**n = 310**
Cancer type, n (%)	
Breast	56 (18.06)
Lung	45 (14.52)
Prostate	27 (8.71)
Colorectal	37 (11.94)
Other^b^	95 (30.65)
Missing data	55 (17.74)
Loco-regional progression of the cancer, n (%)	
Yes	137 (44.19)
No	104 (33.55)
Missing data	69 (22.26)
Sites of metastasis^c^, n (%)	
Brain	9 (2.90)
Bone	113 (36.45)
Lung	60 (19.35)
Lymph node	77 (24.84)
Liver	40 (12.90)
Other	42 (13.55)
Missing data	117 (37.74)
Total number of sites of metastasis, n (%)	
0	35 (11.29)
1	62 (20.00)
2	53 (17.10)
3	24 (7.74)
4	8 (2.58)
5	2 (0.65)
7	1 (0.32)
10	2 (0.65)
Missing data	123 (39.68)

^a^A single patient could have more than one primary diagnoses.

^b^Other cancer types included: sarcoma, myeloma, cervical, pancreatic, renal, bladder, endometrial and head and neck cancer as well as other MedDRA lowest level term types of cancer, including unknown primary cancer.

^c^For site of metastasis more than one response was possible.

CRNP, cancer-related neuropathic pain; MedDRA, Medical Dictionary for Regulatory Activities.

##### Summary of therapeutic management of cancer

The majority of patients with CRNP had received prior or ongoing chemotherapy (62.6%) and/or radiotherapy treatment (53.9%) at the time of the study (Table 6).

**Table 6:** **Therapeutic management of cancer**^**a **^**in patients with CRNP**

	**n = 310**
Chemotherapy, n (%)	
No	55 (17.74)
Yes^b^	194 (62.58)
Radiotherapy, n (%)	
No	79 (25.48)
Yes	167 (53.87)
Prior	138 (44.52)
Ongoing	20 (6.45)
Endocrine (hormone) therapy, n (%)	
No	149 (48.06)
Yes	69 (22.26)
Prior	17 (5.48)
Ongoing	49 (15.81)
Multi-targeted substances, n (%)	
No	167 (53.87)
Yes	39 (12.58)
Prior	15 (4.84)
Ongoing	20 (6.45)
Other, n (%)	
No	91 (29.35)
Yes	40 (12.90)
Prior	7 (2.26)
Ongoing	30 (9.68)

^a^More than one treatment type was possible for each patient.

^b^Chemotherapy treatment included platinum compounds, vinca alkaloids, taxanes, antimetabolites and nitrosoureas among others and treatment could have been before or ongoing at the time of the study.

CRNP, cancer-related neuropathic pain.

##### Summary of recorded surgical treatment

The recorded surgical-procedures for patients with CRNP were varied and included bilateral salpingo-oophorectomy, total abdominal hysterectomy, exeresis, lymphadenectomy and mastectomy. These procedures were categorised according to the nature of the treatment. The number of patients receiving each categorised treatment are as follows (patients could have received more than one): neoadjuvant (cancer treatment given before surgery) = 36 (11.6%) patients; adjuvant (cancer treatment given after surgery) = 67 (21.6%) patients; advanced/metastatic (surgical removal of parts of the body due to secondary cancer) = 29 (9.4%) patients; palliative (surgery to reduce severity of symptoms, etc.) = 17 (5.5%) patients. Ninety-five (30.7%) patients were not given surgical cancer treatments, treatment category data were missing for 39 (12.6%) patients and the surgery CRF page was missing for 56 (18.1%) patients.

#### Health-related quality of life (QOL) in patients with CRNP

##### EQ-5D

The mean health state score on the EQ-5D [16] for patients with CRNP was 51.07 (95% CI 48.39, 53.74) on a scale of 0–100. The mean utility score was 0.53 (95% CI 0.50, 0.56) on a scale of 0–1, with higher score indicating better health. Data were missing for 58 and 60 patients for the health state and utility scores, respectively.

##### SDS

The mean total score on the SDS for patients with CRNP was 18.67 (95% CI 17.50, 19.84) based on functional impairment scales of 0–30 (higher scores indicate greater impairment). Data were missing for 116 patients. Mean scores for the individual items on the SDS are shown in Table 7.

**Table 7:** Individual items of the Sheehan Disability Scale

	**n**	**Mean (SD)**	**95% CI**	**Missing data, n**
Work/school	194	6.74 (3.02)	6.31, 7.17	116
Social life	252	5.94 (3.11)	5.56, 6.33	58
Family life	252	5.90 (2.99)	5.53, 6.27	58
Days lost	208	4.34 (4.63)	3.71, 4.97	102
Days unproductive	206	3.83 (3.03)	3.41, 4.24	104

CI, confidence interval; SD, standard deviation.

#### Patients’ assessment of disease (CRF)

Of the patients with CRNP, 76 (24.5%) recorded no visits to the doctor in relation to the management of neuropathic pain over the past 4 weeks, 180 (58.1%) patients recorded one visit or more and data were missing for 54 (17.4%) patients (Table 8). One hundred forty-one (45.5%) patients with CRNP indicated that their symptoms had an effect on their employment status, while 105 (33.9%) patients with CRNP indicated that they did not. Data were missing for 64 (20.6%) patients (Table 8). The majority (74.8%) of patients with CRNP reported having used prescription medications over past 4 weeks (Table 8).

**Table 8:** Patients with CRNP: assessment of disease

**Items on patient CRF**	**Patients with CRNP n = 310**
Number of visits to a doctor, n (%)	
None	76 (24.52)
1	69 (22.26)
2	64 (20.65)
3	22 (7.10)
≥4	25 (8.06)
Missing data	54 (17.42)
Symptom effect on employment status	
Missing data	64 (20.65)
No	105 (33.87)
Yes	141 (45.48)
Reduced normal life	43 (13.87)
Disabled	56 (18.06)
Unemployed or retired earlier than expected	34 (10.97)
Missing data	8 (2.58)
Treatment used over past 4 weeks^a^	
None	8 (2.58)
Prescription medications	232 (74.84)
Non-prescription medications	27 (8.71)
Physiotherapy	15 (4.84)
Massage	14 (4.52)
Other treatments	23 (7.42)

^a^Patients could give more than one response to this question.

CRF, case report form; CRNP, cancer-related neuropathic pain.

#### Physicians’ evaluation of the PD-Q

Twenty-nine (74.4%) of the surveyed physician population (n = 39) responded yes to the question: “Did you find the painDETECT Questionnaire useful?” Seven of the other 10 physicians responded no and there were missing data for the remaining three physicians. For the question “Did the painDETECT Questionnaire help you evaluate if you think the patient has CRNP?”, of the patients identified to have chronic pain (n=951), physicians responded yes for 334 (35.1%) individual patients and no for 579 (60.9%) patients. Data were missing for 38 (4.0%) patients.

In response to the question “In future would you use the painDETECT Questionnaire?”, none of the physicians indicated that they would use it for all of their patients, 13 (33.3%) indicated that they would for most patients, 15 (38.5%) for some patients and four (10.3%) for a few patients. Five (12.8%) physicians indicated that they would not use it. Data were missing for two physicians.

#### PD-Q end scores and impact on physicians’ clinical assessment

**Figure 2:**
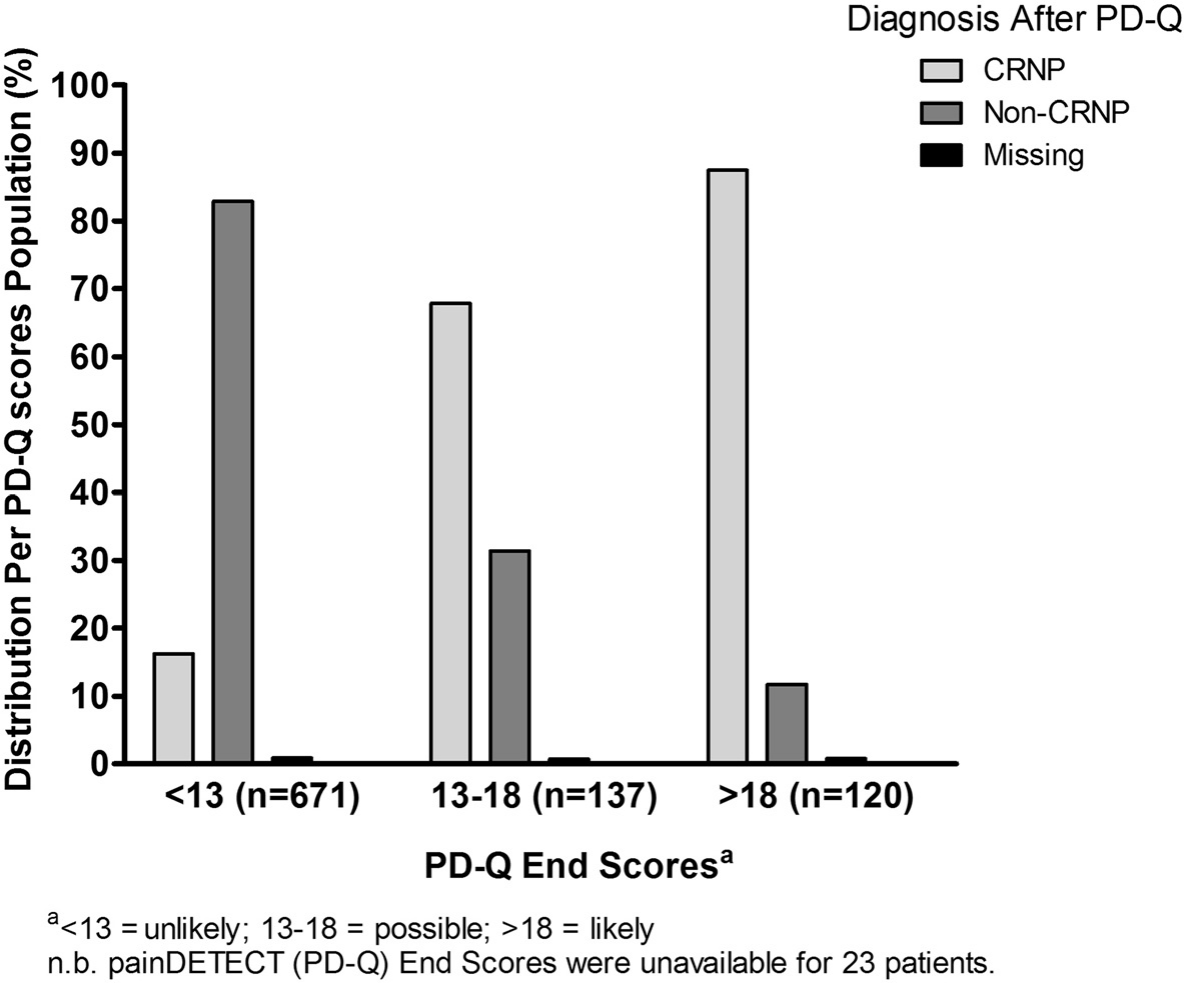
Distribution of participants by PD-Q end scores according to CRNP or non-CRNP diagnosis.

Of the three scoring categories on the PD-Q, the greatest number of patients diagnosed with CRNP scored >18 (likely), followed by scores of 13–18 (possible); the fewest number of patients diagnosed with CRNP scored <13 (unlikely). The opposite trend was apparent in patients without a diagnosis of CRNP (Figure 2).

**Figure 3:**
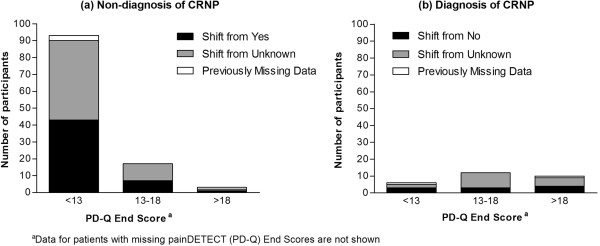
Switch to a negative (a) or positive (b) diagnosis of CRNP, by PD-Q end score.

The shifts in the numbers of patients to either a positive or a negative diagnosis of CRNP following physicians’ examination of the PD-Q (distributed by PD-Q end scores) are shown in Figure 3. For 142 patients, the diagnosis shifted after an examination of the PD-Q; about half (74) of which were previously categorised as unknown. Shifts in patients with a low PD-Q score (<13) were most likely to move towards not having CRNP (93/99), whereas patients with scores >18 (although less in absolute numbers) were more likely to shift towards having CRNP (10/13) in the clinical opinion of the physician. Of the 51 patients who shifted from yes to no, only one (2.0%) had a PD-Q score >18 and of the 10 patients who shifted from no to yes, four (40.0%) patients had a PD-Q score >18.

## Discussion

A total of 951 patients experiencing chronic pain who visited oncology clinics across Europe were enrolled in this study. Approximately 94% of these patients were ≥45 years of age and the ratio of male to female participants was approximately 1.2:1.0. Of the 951 patients with chronic pain, 310 were identified as having CRNP in the opinion of the participating physicians after evaluating the PD-Q. The primary endpoint of this study, the proportion of patients with CRNP in patients with cancer experiencing chronic pain, was therefore 32.6% (95% CI 29.62, 35.58). The percentage of patients with CRNP identified in this study was in line with the international study estimation of 39.7% [18] and within the range of estimates reported in a recent review of studies of neuropathic pain in patients with cancer, which varied from 19% to 39% [4].

Variations in estimates of CRNP may reflect different populations or stages of cancer. In this study, the percentage of patients with CRNP was determined from a sample of adult oncology outpatients experiencing chronic pain. The outpatients with CRNP included in the study presented with varied characteristics and stages of cancer; some experienced loco-regional progression and/or metastasis of the tumour (as shown in the CRNP population), while others had early stage cancer or were cancer survivors. However, the study results are limited by missing data and cannot be generalised to the cancer patient populations as a whole.

Based on available data from the 5882 screened patients, the prevalence point of chronic pain in patients with cancer was estimated at 12.8%. This calculation is lower than previous estimates of chronic pain in patients with cancer at various stages of the disease (including patients who received curative treatment as well as those with advanced stages of the disease) [2]. Reasons for the lower prevalence rate in the current study are not known, but may have been influenced by the criteria for chronic pain that excluded patients who had experienced chronic pain for <3 months or by the screening procedure that excluded patients whose chronic pain was effectively controlled by pain medication. The screening log was intended to enable physicians to identify patients that would have been experiencing chronic pain had they not been receiving pain medications to effectively manage their pain. However, reliably identifying such patients required a complex process, requiring physicians to transfer a particular combination of responses from the screening log to the physician-specific CRF. Missing data, time-constraints and/or oversights resulted in unreliable recording of the number of patients that did not meet the criteria for chronic pain owing to the use of pain medications; therefore, for the purpose of the chronic pain calculations, such patients were excluded. In the opinion of the authors, the number of patients for whom this situation applied would have had little impact on the prevalence point estimate of chronic pain (an underestimation). Some patients also may have failed to report their pain. For example, cancer survivors sometimes fail to acknowledge or report chronic pain because of concerns related to the long-term use of pain-medications [19]. Other reasons that patients with cancer fail to report pain include the desire to be a good patient, a concern that pain is an indication of disease progression, a belief that their physician should focus on treating the cancer rather than the pain or a fear of addiction and/or side effects of pain medications [6,20]. Some of the patients with CRNP may have been experiencing chemotherapy-induced peripheral neuropathy (CINP), which has been documented to cause a diagnostic dilemma for clinicians owing to the range of toxic etiologies that may mimic the clinical features of cancer [21]. Consequently, CINP can go under-reported and under-treated, and may have thereby contributed to the low prevalence point estimate of chronic pain [21].

In the CRNP population, mean m-BPI-sf scores were >4 for each of the individual items (scale of 0–10) with the exception of pain at its least in the last 24 hours (mean = 2.92), indicating that pain in patients with CRNP was either not being effectively managed or was difficult to treat. As noted earlier, there have been reports of general under-treatment of pain in patients with cancer [3,6]. In part, this may be a result of higher priority given to curative treatment compared with effective pain relief or an inadequate knowledge of pain management; for example not tailoring treatment to the specific type of pain [6,20]. In a recent survey conducted in the United States, oncologists identified poor pain assessment as the greatest barrier to managing pain effectively [5].

Average higher scores for each of the three PD-Q pain assessment questions in patients with CRNP compared with the all participants population may indicate that CRNP is more difficult to identify and/or treat effectively than chronic pain without a neuropathic component and that physicians may traditionally choose analgesics primarily effective on nociceptive pain as first-line pain treatment. Indeed, patients with CRNP self-reported mean scores on the EQ-5D and SDS indicated that their symptoms of pain had a negative impact on many aspects of daily functioning and quality of life. Moreover, 45.5% of patients with CRNP indicated that their symptoms had an effect on their employment status, thus highlighting the need for future randomised controlled trials to establish the most effective treatment for neuropathic pain relief within cancer outpatient settings.

The World Health Organization (WHO) guidelines for cancer pain relief state that it is necessary to evaluate the type and level of pain experienced by patients with cancer in order to treat it effectively [22]. In addition to local anaesthetic congeners and/or opioids to treat neuropathic pain or mixed nociceptive and neuropathic pain, the WHO guidelines list tricyclic antidepressants and anticonvulsants as recommended treatments [22]. The European Society for Medical Oncology clinical practice guidelines recommend the treatment of neuropathic pain with both opioid and non-opioid analgesics, in particular tricyclic antidepressants (amitriptyline) or anticonvulsants (gabapentin) [23]. Radiotherapy is recommended for neuropathic pain due to bone metastases [23]. The European Federation of Neurological Societies Task Force guidelines for neuropathic pain assessed the evidence for gabapentin as a treatment for CRNP as level A and amitriptyline and tramadol as level B (based on a single study) [24]. A recent review of treatments for cancer pain listed multi-purpose analgesics (i.e. glucocorticoids and tricyclic antidepressants) and the anticonvulsants gabapentin and pregabalin as effective treatments for neuropathic pain in patients with cancer [25].

The most commonly prescribed treatments for neuropathic pain in the CRNP population in this study were non-opioid analgesics, a strong opioid and/or anticonvulsants. It should be noted that these patients also may have been receiving more than one therapeutic treatment and/or demonstrated a mixed pain profile. Of the available data in the physician study assessments, mixed cancer-related pain with a neuropathic component was evident in 47.1% of patients with CRNP and in 32.3% of those with pure CRNP. Data were missing for 64 (20.6%) patients with CRNP. Neuropathic pain was considered to be directly due to the tumour in 63.6% of patients with CRNP and due to cancer treatment in 29.0%. However, the types and characteristics of cancer in patients with CRNP in this study were varied, as were their experiences of therapeutic management and surgical treatment of the disease. These data highlight that neuropathic pain is not specific to patients with a particular cancer and/or treatment profile and demonstrate the need to screen for neuropathic pain in all patients with cancer, regardless of the stage or type of disease.

A further objective of this study was to assess the usefulness of the PD-Q as a screening tool to help physicians identify neuropathic pain in patients with cancer. The usefulness of PD-Q for detecting neuropathic cancer pain has been previously assessed in comparison to the Edmonton Classification System of Cancer Pain (ECS-CP). Based on their initial categorization of patients as having a neuropathic component if their PD-Q score ranged from 13 to 38 (similar to our study) and the comparison to the ECS-CP, the authors calculated the sensitivity and specificity of PD-Q for the detection of cancer-related neuropathic pain as 53% and 77%, respectively [26]. In the current study, the distribution of patients with and without a diagnosis of CRNP according to the PD-Q end-scores was largely in keeping with the physicians’ assessment of the likelihood that a patient is experiencing neuropathic pain. There is evidence that the use of the PD-Q changed physicians’ clinical opinions in some cases, in particular, the shift to a diagnosis of not having CRNP for patients with PD-Q end scores of <13. Many patients for whom physicians changed their initial opinion to a positive diagnosis of CRNP were those whose diagnosis was recorded as unknown before evaluation of the PD-Q. Thus, the PD-Q may be a useful tool to help identify CRNP in situations in which physicians are initially unsure.

The majority (74.4%) of the 39 physicians who completed the physician-specific CRF indicated that they found the PD-Q a useful tool to help detect CRNP in daily practice and over 70% indicated that they would use this tool to evaluate most, or some, of their patients in the future. The PD-Q was initially developed to identify neuropathic pain in patients with back pain as a model for mixed pain conditions [14], and, as with many other screening tools, it has not been exclusively validated for use in patients with cancer. However, these results suggest that the PD-Q may be a useful screening tool for CRNP.

As with all non-interventional studies, there are particular advantages and limitations of the design of this study. This large-scale multi-centre study allowed the opportunity to collect epidemiological data from patients with cancer across Europe and add to the literature relating to CRNP. The limitations associated with this study include the difficulties in recruiting investigators who were willing to participate. This proved more challenging than anticipated and impacted the enrolment of participants, thereby leading to a decrease in the planned sample size. Incomplete or missing CRF forms also were a consequence of the non-interventional methodology that may have impacted on the overall chronic pain prevalence estimates, which should therefore be interpreted with caution.

## Conclusions

This observational, non-interventional, cross-sectional, multi-centre study showed that a proportion of adult patients with cancer who were attending outpatient clinics in Europe were experiencing uncontrolled pain and almost a third had neuropathic pain. The results of this study highlight the wide range of patients with cancer experiencing CRNP and emphasise the importance of detecting neuropathic pain in order to treat it effectively. Screening tools like the PD-Q may help physicians identify neuropathic pain in patients with cancer for the purposes of effective pain management, which may ultimately impact the functional ability and quality of life of patients with CRNP.

## Abbreviations

CI: Confidence interval; CRF: Case report form; CRNP: Cancer-related neuropathic pain; EuroQOL: EQ-5D Health questionnaire; m-BPI-sF: Modified Brief Pain Inventory Short Form; PD-Q: PainDETECT questionnaire; QOL: Quality of life; SDS: Sheehan Disability Scale; WHO: World Health Organization.

## Competing interests

Financial competing interests

C. Garzón-Rodríguez has disclosed acting as a consultant/advisor for Pfizer. L. Olay Gayoso, J. Sepúlveda, E. Samantas and U. Pelzer have declared no conflict of interest. L. Lyras, C. van Litsenburg and M. Strand are all employees of Pfizer, own Pfizer Stock and have Pfizer stock options. S. Bowen, previously an employee of Pfizer, was a paid consultant to Pfizer and owns Pfizer stock and has Pfizer stock options. The authors were not compensated for their work on the manuscript.

Non-financial competing interests

The authors have no political, personal, ideological, academic, intellectual, or commercial competing interests in relation to this manuscript, other than the financial competing interests declared above.

## Authors’ contributions

CGR, LOG, JMS, ES and UP participated in the conduct of the study and contributed to data collection at one or more study site. They each contributed to the interpretation of the data and were involved in drafting and reviewing the manuscript for intellectual content. LL, CvL and MS participated in the design and conduct of the study, interpretation of the data and were involved in drafting and reviewing the manuscript for intellectual content. SB conducted the statistical analysis, and participated in the interpretation of data, writing and reviewing the manuscript. All authors discussed the results, commented on the manuscript, reviewed and approved the final version of the manuscript for publication.

## Pre-publication history

The pre-publication history for this paper can be accessed here:

http://www.biomedcentral.com/1472-684X/12/41/prepub
